# Non-High-Density Lipoprotein Cholesterol Levels on the Risk of Asymptomatic Intracranial Arterial Stenosis: A Result from the APAC Study

**DOI:** 10.1038/srep37410

**Published:** 2016-11-18

**Authors:** Jianwei Wu, Anxin Wang, Xin Li, Shouling Wu, Xingquan Zhao

**Affiliations:** 1 Department of Neurology, Beijing Tiantan Hospital, Capital Medical University, Beijing, China; 2 China National Clinical Research Center for Neurological Diseases, Beijing, China; 3 Center of Stroke, Beijing Institute for Brain Disorders, Beijing, China; 4 Beijing Key Laboratory of Translational Medicine for Cerebrovascular Disease, Beijing, China; 5 Department of interventional neurology, The affiliated hospital of Qingdao university, Qingdao, China; 6 Department of Cardiology, Kailuan Hospital, Hebei United University, Tangshan, China

## Abstract

Intracranial arterial stenosis (ICAS) is an important cause of ischemic stroke and transient ischemic attack (TIA), and the correlation between the plasma non-high density cholesterol (non-HDLC) levels and ICAS, especially asymptomatic ICAS (AICAS) is not clear. The Asymptomatic Polyvascular Abnormalities Community(APAC) study is a community-based, prospective, long-term follow-up observational study. 3387 participants were enrolled in this study. The diagnosis of AICAS was made by transcranial Doppler ultrasonography. The participants were then divided into 3 groups based on their non-HDLC levels. The cox regression was used to analyze the correlation between the non-HDLC level and the incidence of AICAS.9.98% of the participants were diagnosed with AICAS during 2 years following up. Multivariate analysis showed that non-HDL-C is an independent indicator for the incidence of AICAS (HR = 1.22, 95%CI: 1.06–1.40), The incidence of AICAS gradually increase with the increasing non-HDLC level. Compared with subgroup(non-HDLC < 3.4 mmol/l), incidence of AICAS was significantly higher in the subgroups(non-HDLC 3.4–4.1 mmol/l and non-HDLC ≥ 4.1 mmol/l) after adjustment for the confounding factors (HR = 1.32, 95%CI:1.02–1.73; HR = 1.46, 95%CI: 1.10–1.94, respectively). In conclusions, our findings suggest that elevated non-HDLC levels a significant risk factor for the development of AICAS in the APAC study.

Intracranial arterial stenosis (ICAS) is an important cause of ischemic stroke and transient ischemic attack (TIA)^1^,^2^. The 1-year recurrence rate of TIA or ischemic stroke is 23% in patients with ICAS of more than 70%^3^. In contrast to extracranial atherosclerosis, intracranial atherosclerosis occurs more commonly in Asian^4^. In China, about 30–40% of ischemic strokes and over 50% of transient ischemic attacks (TIA) are associated with the presence of ICAS^5^. Therefore, it is important to screen for the risk factors of asymptomatic intracranial arterial stenosis (AICAS) and through early interventions to decrease the risk of stroke. In the past study, we demonstrated that non-high density cholesterol (non-HDLC) is an independent predictor of AICAS prevalence^6^, but it was a cross-sectional study, we were not able to evaluate the effect of non-HDLC on the development of intracranial atherosclerosis. We did not know if the higher level of non-HDLC risk were present before or after the development of ICAS and the real cause-and-effect relationship between the two. As the study is ongoing, here we reported a large prospective cohort study with Chinese population to test if non-HDLC is associated with risks of AICAS.

## Results

### Incidence of ICAS

During the follow up, 338 patients developed ICAS based on TCD results, representing the incidence of 9.98% (338/3387).

### Baseline characteristics

Subjects’ baseline characteristics are presented in Table 1. The mean values of the baseline characteristics are presented for the non-HDLC levels subgroups. Non-HDLC mean values were 2.73 mmol/l, 3.72 mmol/l, and 4.79 mmol/l for subgroups 1 to 3, respectively. There were significant differences for age, BMI, waist circumference, total cholesterol, HDL-C, TC/HDLC and triglycerides among subgroups (P < 0.01). Absolute values of all these variables, with the exception of HDL-C, continuously increased as non-HDLC levels increased. In contrast, serum HDL-C levels were lower with higher levels of non-HDLC. A larger proportion of subjects with higher non-HDLC levels had concomitant hypertension, metabolic syndrome and smokers. In contrast, a larger proportion of subjects were men and suffered diabetes in subgroup 3.4 mmol/l-4.1 mmol/l.

Correlation between baseline non-HDLC levels and the incidence of AICAS. As shown in Table 2, Compared with the first subgroup (<2.6 mmol/l), incidence of asymptomatic ICAS was significantly higher in the second and third subgroup(2.6–3.4 mmol/l and >3.4 mmol/l) after adjustment for the confounding factors (HR = 1.32, 95%CI: 1.02–1.73; HR = 1.46), 95%CI: 1.10–1.94, respectively). We observed that non-HDLC is an independent indicator for the incidence of AICAS(multivariate-adjusted (HR = 1.22, 95%CI: 1.06–1.40).

When baseline characteristics (including sex, age, BMI, hypertension, diabetes, and smoking status) were evaluated, our results indicated that the presence or absence of these indicators did not influence the association between non-HDLC levels and the incidence of A ICAS (P = 0.1372, 0.7809, 0.2450, 0.8071, 0.7240 and 0.8233 respectively) (Table 3).

## Discussion

In this study, we found that non-HDLC was an independent indicator for the incidence of asymptomatic ICAS. To the best of our knowledge, this is the first evidence specifically showing the significant association between non-HDLC levels and the incidence of asymptomatic ICAS in a prospective study.

In the baseline, we also found that non-HDLC is an independent indicator for the presence of ICAS (OR = 1.15, 95%CI: 1.08–1.23). Compared with the first quintile, multivariate-adjusted OR (95%CI) of the second, third, fourth and fifth quintiles were: 1.05 (0.71–1.56), 1.33 (0.91–1.95), 1.83 (1.27–2.63), 2.48 (1.72–3.57), respectively^6^.

Non-HDLC is a composite marker of several atherogenic lipoproteins, including LDL, very-low-density lipoprotein (VLDL), intermediate-density lipoprotein (IDL), and lipoprotein(a)^7^. Serum non-HDLC measures all atherogenic apolipoprotein B-containing lipoproteins, compared with LDL-C, non–HDLC contains a higher degree of atherogenic lipoproteins.

Most clinical guidelines acknowledge the central role of LDL-C in atherosclerosis and it is considered a key parameter in CVD risk estimation and a primary target in CVD prevention^8^,^9^. However, there is accumulating evidence that other lipid measures such as non-HDLC may be more accurate predictors of CVD risk. Although LDL-C has traditionally been the primary target of therapy, the NLA Expert Panel’s consensus view is that non-HDLC is a better primary target for modification than LDL-C^10^.

Growing evidence suggests that non-HDLC is a stronger predictor for coronary heart disease (CHD) than LDL-C. In 1996, the Systolic Hypertension in Elderly Program was among the first studies to describe an association between non-HDLC levels and coronary heart disease(CHD) risk^11^. The relative risk (RR) for CHD per standard deviation (SD) increase was 1.32 (95% CI 1.13–1.54) for non-HDLC and 1.19 (95% CI 1.02–1.39) for LDL-C. In the Women’s Health Study, which enrolled 15 632 women aged 45 years or over, non-HDLC was associations with the risk of future cardiovascular events, which was stronger than for LDL-C^12^. Adjusted RRs for a comparison of extreme quintiles were 2.51 (95% CI 1.69–3.72) for non-HDLC and 1.62 (95% CI 1.17–2.25) for LDL-C. Among 46 786 patients with type 2 diabetes mellitus, HRs for top versus bottom quartiles were 1.95 (95% CI 1.74–2.19) for non-HDLC and 1.63 (95% CI 1.46–1.82) for LDL-C^13^. In a meta-analysis among 233 455 people, the relative risk ratio (RRR) for ischemic cardiovascular events per 1-SD increment was 1.34 (95% CI 1.24–1.44) for non-HDLC, which was substantially stronger than 1.25 (95% CI 1.18–1.33) for LDL-C^14^.

Studies also found that non-HDLC is an important predictor for ischemic stroke as our study. The Kailuan study including 95 778 participants, found that serum non-HDLC level is a stronger predictor for the risk of ischemic stroke than serum LDL-C level in the Chinese population. The hazard ratio(HR) for ischemic stroke in the top quintile was 1.53 (95% CI, 1.24–1.88) for non-HDLC, which was substantially stronger than 1.25 (95%CI, 1.01–1.53) for LDL-C^15^. The Hisayama Study, including 2452 community-dwelling Japanese subjects aged >40 years, evaluated the association between non-HDLC levels and the risk of type-specific cardiovascular disease in the general Japanese population. After adjustment for confounders, the associations remained significant for atherothrombotic infarction (adjusted hazard ratio (HR) for a 1 standard deviation of non-HDLC concentrations = 1.39, 95% CI = 1.09 to 1.79)^16^. A study in chinese, including 27,020 participants aged 35 to74 years found that an increase of 30 mg/dl in non-HDLC level would correspond to 12% increase in risk of stroke^17^.

Possible explanations for the superiority of non-HDLC over LDL-C for predicting ASCVD event risk include: (1) as with LDL, some triglyceride-rich lipoprotein remnants enter the arterial wall, and thus contribute to the initiation and progression of atherosclerosis, (2) non-HDLC correlates more closely than LDL-C with apo B, thus more closely correlates with the total burden of atherogenic particles, and (3) elevated levels of triglycerides and VLDL-C reflect hepatic production of particles with greater atherogenic potential, such as those having poor interactivity with hepatic receptors, resulting in longer residence time in the circulation.

### Limitations

There were some limitations in our study despite of the careful study design. First, all participants with poor temporal window reading in TCD were considered non-ICAS in our study. This might result in a significantly underestimated incidence of ICAS. Second, our study was based on a randomly selected subgroup of participants of the Kailuan Study that included employees and retirees of the Kailuan Co. Ltd. and the study population was selected using a stratified random sampling method by age and sex. It may not be representative of the population of the Tangshan area in Hebei province despite the large study sample. Third, 1233 participants were missing TCD during follow-up, which may lead to the results migration. Despite these limitations, our study was so far the first large, prospective clinic trial to our knowledge that investigate the correlation between non-HDLC level and asymptomatic ICAS.

### Future directions

More epidemiological and experimental data are needed to further confirm the correlation between non-HDLC level and asymptomatic ICAS in future.

In conclusion, our findings suggest that elevated non-HDLC levels a significant risk factor for the development of AICAS in the APAC study.

## Subjects and Methods

### Study Population

The Asymptomatic Polyvascular Abnormalities Community (APAC) study is a community-based, prospective, long-term follow-up observational study, aiming to investigate the epidemiology of asymptomatic polyvascular abnormalities in Chinese adults. A detailed description of this study has been published previously^6^,^18^,^19^,^20^. The APAC study included 5440 participants. For participants who took the every two-year routine medical examination, they were followed up by face-to-face interviews by hospital physicians and nurses. During the baseline survey and the every two-year routine medical examination, the participants underwent extensive clinical examination, laboratory tests and transcranial Doppler (TCD) examinations. In this study, the following participants were excluded: 75 participants who had taken lipid-lowering agents; 14 participants with incomplete non-HDLC data; 698 participants who had intracranial arterial stenosis (ICAS) at baseline; 1234 participants who miss to TCD during the follow-up; 32 participants who suffered TIA or stroke during the follow-up. Eventually, a total of 3387 participants were included in the analyses. The study was performed according to the guidelines from the Helsinki Declaration and was approved by the Ethics Committees of the Kailuan General Hospital and the Beijing Tian Tan Hospital. Written informed consent was obtained from all participants.

### Measurement of Indicators

A questionnaire was used to obtain information on enrolled subjects, including age, gender, hypertension, diabetes, smoking, and medications prescribed by physicians. Smoking status was classified into “non-smoking” and “smoking” according to self-reported information. Hypertension was defined based on: personal history of hypertension, a systolic blood pressure ≥140 mmHg, a diastolic pressure ≥90 mmHg, or currently taking antihypertensive medication prescribed by a physician. Subject height was measured and body mass index (BMI) was calculated as body weight (kg) divided by the squared height (m2). Diabetes mellitus was diagnosed if the subject was undergoing treatment with insulin or oral hypoglycemic agents, if fasting blood glucose (FBG) levels were ≥126 mg/dl, or if he had a personal history of diabetes mellitus. Non-HDLC levels were determinedby subtracting serum HDLC levels from total cholesterol^21^.

TCD was performed by two experienced neurologists using portable devices (EME Companion, Nicolet).ICAS diagnosis were defined by a peak systolic flow velocity of: >140 cm per second for the middle cerebral artery >120 cm per second for the anterior cerebral artery >100 cm per second for the posterior cerebral artery and vertebra-basilar artery, and >120 cm per second for the siphon internal carotid artery.

In addition to the above criteria, patients, age, presence of disturbance in echo frequency, turbulence and whether the abnormal velocity was segmental were also taken into consideration for ICAS diagnosis^22^. Subjects without a good temporal window were considered without stenosis. Patients were classified as having occlusive disease if at least one of the studied arteries showed evidence of stenosis or occlusion.

### Statistical analysis

We classified the participants into 3 groups according to serum non-HDL-C levels(group1: <3.4 mmol/l group2: 3.4–4.1 mmol/l group3: ≥4.1 mmol/l)^23^. Continuous variables were compared using analysis of variance (ANOVA) and categorical variables were compared using chi-square tests. The age- and gender-adjusted or multivariate-adjusted hazard ratios (HRs) and 95%CI were calculated using cox regression models. The multivariate-adjusted model was further adjusted for age, gender, BMI, hypertension, diabetes, current smoking status, HDL-C levels and triglycerides. Additionally, gender and other potential indicators were also evaluated to assess if there was any significant interaction between these variables and the relationship between non-HDLC levels and AICAS incidence. All statistical analyses were performed using the SAS program package. A P-value < 0.05 was considered statistically significant.

## Additional Information

**How to cite this article**: Wu, J. *et al*. Non-High-Density Lipoprotein Cholesterol Levels on the Risk of Asymptomatic Intracranial Arterial Stenosis: A Result from the APAC Study. *Sci. Rep*. **6**, 37410; doi: 10.1038/srep37410 (2016).

**Publisher's note**: Springer Nature remains neutral with regard to jurisdictional claims in published maps and institutional affiliations.

## Figures and Tables

**Table 1:** Mean and prevalence as baseline characteristics of participants according to non-HDLC.

Non-HDLC, mmol/l				
	<3.4	3.4–4.1	≥4.1	P
Mean (SD), mmol/l	2.73(0.48)	3.72(0.20)	4.79(0.78)	
number	1837	837	713	
Age, year	52.0(10.4)	55.5(10.9)	55.5(10.2)	<0.0001
Female, %	45.4	39.3	42.9	0.0135
Smoking, %	34.0	38.0	40.4	0.0052
hypertension, %	17.8	26.1	26.2	<0.0001
diabetes, %	5.66	7.89	6.87	0.0842
PAD, %	2.18	2.27	2.52	0.2786
Metabolic syndrome, %	15.2	31.3	37.6	<0.0001
BMI, kg/m2	24.5(3.14)	25.2(3.21)	25.5(3.26)	<0.0001
Waist circumference, cm	84.1(9.80)	86.8(9.54)	88.0(9.82)	<0.0001
Glucose, mmol/l	5.33(1.22)	5.57(1.37)	5.77(1.65)	<0.0001
TC, mmol/l	4.41(0.57)	5.29(0.45)	6.35(0.87)	<0.0001
HDLC, mmol/l	1.68(0.42)	1.57(0.31)	1.56(0.40)	<0.0001
TC/HDLC	2.74(0.58)	3.54(0.78)	4.29(1.10)	<0.0001
TG, mmol/l	1.30(0.87)	1.77(1.19)	2.51(2.36)	<0.0001

**Table 2:** HRs of ICAS for various levels of lipids measured at baseline.

Non-HDLC	Case (n)	HR	Medol1 (95%Cl)	Medol2 (95%Cl)
<3.4	152(8.3%)	1	1	1
3.4–4.1	97(11.6%)	1.39(1.08–1.79)	1.29(1.00–1.67)	1.32(1.02–1.73)
≥4.1	89(12.6%)	1.44(1.11–1.87)	1.32(1.01–1.72)	1.46(1.10–1.94)
P for trend		P < 0.01	P < 0.01	P < 0.01
Continuous Scale		1.21(1.07–1.38)	1.16(1.02–1.32)	1.22(1.06–1.40)

HR, hazard ratio; 95% CI., 95% confidence interval.

Model 1: adjusted for age, sex.

Model2: adjusted for age, sex, bmi, hypertension, diabetes smoke, HDLC, TG by a Cox proportional hazard model.

**Table 3:** HR of ICAS According to Non-High-Density Lipoprotein Cholesterol Levels, Stratified According to Selected Risk Factors

	<3.4	3.4–4.1	≥4.1	P for interaction
Sex				0.1372
male	1	1.04(0.70–1.53)	1.08(0.71–1.66)	
female	1	1.67(1.17–2.38)	2.01(1.36–2.96)	
Age				0.7809
≥60	1	1.10(0.66–1.85)	1.50(0.90–2.52)	
<60	1	1.45(1.07–1.94)	1.48(1.05–2.09)	
BMI, kg/m2				0.2450
<25	1	1.42(0.99–2.01)	1.68(1.33–2.48)	
≥25	1	1.18(0.80–1.75)	1.23(0.81–1.86)	
Hypertension				0.8071
No	1	1.34(0.99–1.81)	1.39(0.99–1.97)	
Yes	1	1.27(0.75–2.16)	1.60(0.95–2.71)	
Diabetes				0.7240
No	1	1.29(0.97–1.70)	1.40(1.04–1.89)	
Yes	1	1.75(0.76–4.06)	1.89(0.72–4.99)	
Smoking				0.8233
No	1	1.37(1.00–1.88)	1.57(1.11–2.22)	
Yes	1	1.18(0.72–1.91)	1.20(0.72–1.99)	

Multivariate-adjusted hazard ratio (HR): adjusted for age, sex, bmi, hypertension, diabetes smoke, HDLC, TG by a Cox proportional hazard model.
